# Squamous Cell Cancer Arising in an African American Male Cheek from Discoid Lupus: A Rare Case and Review of the Literature

**DOI:** 10.1155/2016/9170424

**Published:** 2016-08-17

**Authors:** Emanuel A. Shapera, Paul D. Kim

**Affiliations:** ^1^Department of General Surgery, University of New Mexico (UNM) School of Medicine, MSC 10 5610, 1 University of New Mexico, Albuquerque, NM 87131, USA; ^2^Department of Head & Neck Surgery, Kaiser Fontana/Ontario, 9961 Sierra Avenue, Fontana, CA 92335, USA

## Abstract

A 50-year-old African American male with Discoid Lupus Erythematosus (DLE) presented to the dermatology clinic for a rapidly enlarging left cheek mass. The mass failed to resolve with conservative measures. A biopsy revealed poorly differentiated Squamous Cell Carcinoma (SCC). He was referred to Head and Neck Surgery and successfully underwent a resection with free flap reconstruction. Postoperatively he did well. Squamous cell skin carcinomas arising from lesions of Discoid Lupus are rare and aggressive tumors with greater likelihood of metastases. Cases have been reported among patients with different clinical characteristics; we present a rare case arising in an African American male on the face and involving the ear.

## 1. Introduction

Squamous Cell Carcinoma can arise from lesions of DLE as a long-term complication. Purported associations include HPV infection, immunosuppressive drug regimens, and exposure to sunlight. These lesions are rare, aggressive, and tend to metastasize more frequently than the more “traditional” squamous cell skin carcinoma arising from actinic keratosis. Consequently the best chance for cure is aggressive surgical excision ensuring negative margins and removal of adenopathy. Cases have been reported in individuals of varying ethnicities with different predispositions to skin cancer, but none have been published until now arising from an African American male on the left cheek and ear.

## 2. Case Report

**Figure 1:**
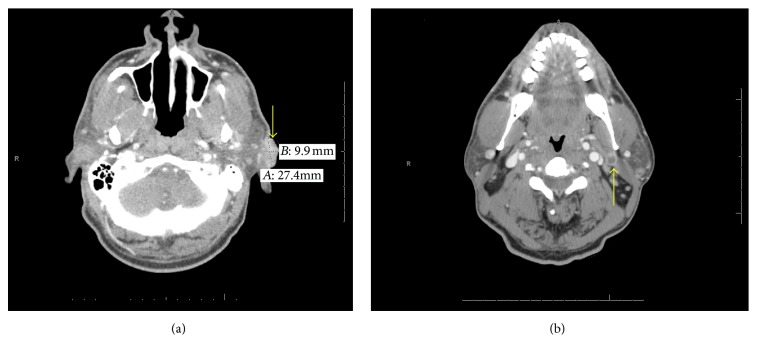
CT scan demonstrating primary tumor within the left cheek (a) and a necrotic lymph node (b).

A 50-year-old African American male with a past medical history significant for Insulin Independent Diabetes Mellitus and a 20-year history of DLE presented to the dermatology clinic with a rapidly enlarging left cheek and ear mass. He underwent two shave biopsies, initially nondiagnostic followed by a subsequent biopsy revealing poorly differentiated SCC. He was then referred for surgery. Preoperative imaging revealed a left facial mass with adjacent ear canal invasion with metastatic adenopathy (Figures 1(a) and 1(b)). After informed consent, he successfully underwent a radical excision of left ear and face, left total parotidectomy, left modified neck dissection, left lateral temporal bone resection, and anterolateral thigh free flap for reconstruction. Final pathology revealed a well-differentiated Squamous Cell Carcinoma with multiple levels of neck adenopathy. There was direct invasion from the skin into the parotid, ear cartilage, and bone but no perineural or vascular invasion identified. Postoperatively the patient healed without any major complications and was referred for adjuvant radiation therapy. Unfortunately, the patient developed recurrence in the ipsilateral axilla and contralateral neck lymph nodes and the patient was referred to palliative care.

## 3. Discussion

Data from literature reporting on SCC arising from DLE is limited. One of the largest studies is a case series in China involving 58 patients with DLE complicated by Squamous Cell Carcinoma [1]. The authors of the study, Tao et al., cited an incidence of approximately 3% from two studies by Millard et al. and de Berker et al. Millard's study initially reported that 2/38 patients with 5 years of DLE had Squamous Cell Carcinoma, a 5.26% incidence.

Risk factors for Squamous Cell Carcinoma arising from Discoid Lupus include tobacco use; in Tao et al.'s study, the majority of tumors occurred in the lower lip (66%), followed by the cheeks [1]. Other sites included the forearms and the back of hands. These sites are exposed to the sun, and like conventional Squamous Cell Carcinoma of the skin, cancer originating from DLE increases with sun exposure. Not coincidentally, a larger percentage of individuals with lower lip malignancy smoked (31.6%) compared to individuals with tumors at all other sites (5.3%), a statistically significant difference (*p* < 0.0418). This suggests that smoking cessation should play a major role in prevention. The time to develop lower lip tumors from the onset of DLE is shorter than for tumors at other sites. Patients develop lower lip malignancy on average 12.4 years into their diagnosis as opposed to 19.2 years for patients with tumors at other sites, a statistically significant difference (*p* < 0.01). In spite of this, Parikh et al. reported a case of SCC arising from a DLE lesion that was 1 year old, although the patient had a 19-year history of connective tissue disease and a SCC on the chest excised 6 years earlier [2], suggesting that the patient may have had undiagnosed prior disease. Otherwise no literature suggests malignancy arising sooner than 5 years from onset of DLE. This might prove useful in tailoring intensive screening for malignancy towards individuals who have had DLE for at least a few years.

In comparison to “conventional” SCC, Tao et al. reported a higher percent of recurrence (29% versus 20%), metastases (16.1% versus 0.5–6%), and death (19.4% versus 1%). A 100% mortality rate was reported by the same study for patients with metastatic SCC as opposed to 30% of patients with conventional metastatic SCC, but it is not specified whether this is disease specific or overall mortality [1]. In Sherman et al. study of 10 African American patients with SCC arising from DLE lesions, 3 out of 4 patients with metastatic SCC died [3]. In contrast, Sulica and Kao mentioned that 2 out of 6 patients with metastatic disease had died out of a total of 19 patients with SCC [4]. Neither of these studies defined their follow-up period. Our patient presented with 4 lymph nodes positive for metastasis and invasion into cartilage and bone, but with surgical excision to negative margins.

**Figure 2:**
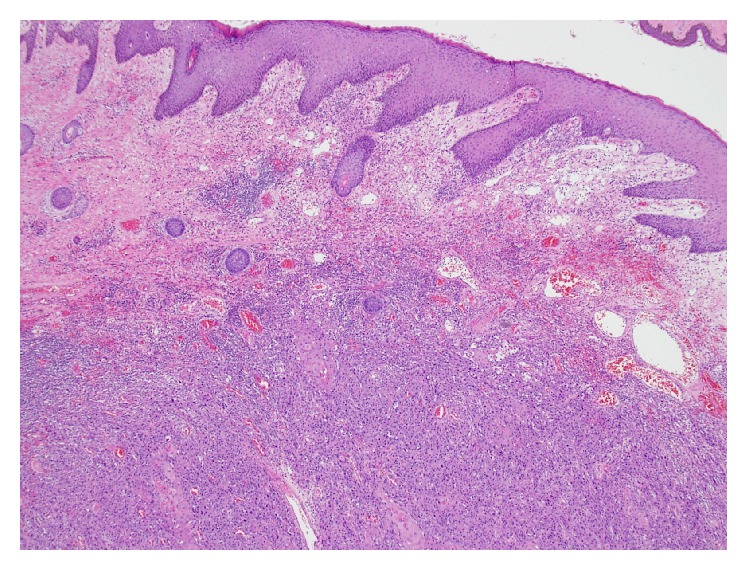
Pathologic slide demonstrating normal dermis (superior), inflammatory infiltrate (inferiorly), and well-differentiated Squamous Cell Carcinoma (bottom).

**Figure 3:**
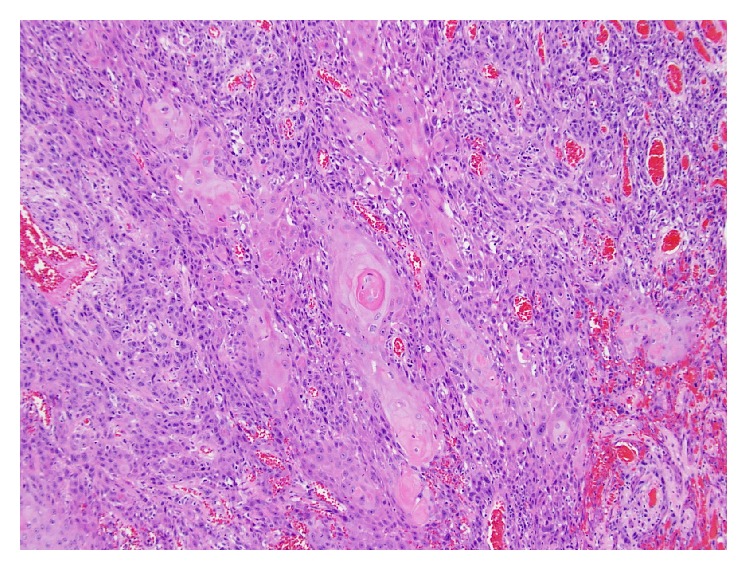
Higher magnification of Figure 2 demonstrating invasive, well-differentiated Squamous Cell Carcinoma with characteristic “whorl” pattern, center.

Histopathological assessment can present an additional challenge in managing these lesions. Dhingra et al. reported a case where a patient's initial biopsy of a discoid lesion of the lip was read as benign 12 months prior to becoming a clinically evident tumor [5]. The difficulty lies in distinguishing hypertrophic discoid lesions from SCC. Both Dhingra et al. [5] and Parikh et al. [2] studies discuss a hyperplastic basal membrane with mononuclear infiltrate as being suggestive of benign hypertrophic lupus erythematosus (HLE). Keratinocyte atypia and irregular elongation, in contrast, present in carcinoma (Figures 2 and 3). In cases reported by Harper et al. and Alsanafi and Werth, patients had initial biopsies read as benign, with the latter study highlighting one case where a patient had 2 biopsies read as negative with a potential 3-year delay in diagnosis before a third biopsy was positive for tumor [6, 7]. While it is possible that tumor may have developed in the interim between these biopsies, it raises the concern and need for expert histopathologic interpretation to prevent missing an aggressive tumor. In one of the cases reported by Alsanafi and Werth, the patient initially had no lymph nodes tumor invasion, though subsequently after resection the nodes were found to be positive, eventually requiring a mid-arm amputation [7]. Vinciullo suggested the use of immunofluorescence to assist in distinguishing SCC from HLE [8]. The investigators in the study by Simpson et al. discussed the association and likely worsening aggression of SCC in patients infected with HPV [9]. Immunochemistry detecting HPV proteins in a biopsy could facilitate the pathological diagnosis of SCC.

Cutaneous SCC is likelier to occur in individuals with lighter skin color. Cases have been reported with SCC arising from DLE lesions in the extremities, the ears, the groin, and different ethnicities, but we present a rare report of a tumor arising just below the left ear in the cheek of an African American male.

## 4. Conclusion

Our patient had DLE for 20 years. The patient presented with two previous negative biopsies, yet at the time of diagnosis of malignancy the patient had multiple metastatic lymph nodes. As SCC arising from DLE lesions have worse outcomes in terms of metastasis, recurrence, and death compared to conventional squamous cell cancer of the skin, a thorough dermatologic search for tumors in all patients who have had Discoid Lupus Erythematosus for 5 years or more should be performed routinely by physicians, including repeat biopsies and close follow-up for any worsening lesions treated with antilupus therapy. Treatment of SCC should involve radical excision with generous margins due to the elevated risk of recurrence, difficulty in obtaining a histopathological confirmation, and the poor outcome in recurrence and metastasis. The best course of action for all practitioners is to promote the modification of identifiable risk factors: the prevention of smoking, protection against ultraviolet radiation, and the vaccination against oncogenic strains of HPV. The prognosis for our patient remains poor.
